# *In vitro* antioxidant, anti-inflammatory and anticancer activities of ethyl acetate soluble proanthocyanidins of the inflorescence of *Cocos nucifera* L.

**DOI:** 10.1186/s12906-016-1335-2

**Published:** 2016-09-05

**Authors:** Chayanika Padumadasa, Durga Dharmadana, Ajit Abeysekera, Mayuri Thammitiyagodage

**Affiliations:** 1 Department of Chemistry, Faculty of Applied Sciences, University of Sri Jayewardenepura, Nugegoda, Sri Lanka; 2 Animal Centre, Medical Research Institute, Colombo 8, Sri Lanka

**Keywords:** *Cocos nucifera*, inflorescence, Proanthocyanidins, Ethyl acetate extract, Antioxidant, Anti-inflammatory, Anticancer

## Abstract

**Background:**

Proanthocyanidins belong to a class of polyphenolic compounds called flavonoids and have been reported to exhibit important biological activities. The immature inflorescence of *Cocos nucifera* L. is used by Ayurvedic and traditional medical practitioners for the treatment of menorrhagia in Sri Lanka. Our studies have shown that the inflorescence of *Cocos nucifera* L. predominantly contains proanthocyanidins.

**Objective:**

To determine the antioxidant, anti-inflammatory and anticancer activities of ethyl acetate soluble proanthocyanidins (EASPA) of immature inflorescence of *Cocos nucifera* L.

**Methods:**

EASPA fraction of an acetone/water (7:3) extract of *Cocos nucifera* L. inflorescence was purified on Sephadex LH-20 and was used for the study. Antioxidant activity of EASPA was determined using DPPH and SOR scavenging assays. Anti-inflammatory activity of EASPA was determined by oxidative burst assay using chemiluminescence technique. MTT colorimetric assay was used to evaluate the cytotoxicity of EASPA to both PC3 and HeLa cells.

**Results:**

EASPA showed radical scavenging activity against both DPPH and superoxide radicals with IC50 values of 11.02 ± 0.60 μg/mL and 26.11 ± 0.72 μg/mL. In both assays, EASPA showed less antioxidant activity than the standards used. It exhibited similar anti-inflammatory activity (IC50 = 10.31 ± 1.11 μg/mL) to ibuprofen (IC50 = 11.20 ± 1.90 μg/mL) (P ≥ 0.05). EASPA also showed stronger cytotoxic activity towards Hela cells (IC50 = 18.78 ± 0.90 μg/mL) than tamoxifen (IC50 = 28.80 ± 1.94 μg/mL) (*P* ≤ 0.05), while low cytotoxicity was observed against PC3 cells (IC50 = 44.21 ± 0.73 μg/mL) compared to doxorubicin (IC50 = 1.38 ± 0.16 μg/mL).

**Conclusion:**

EASPA showed antioxidant, anti-inflammatory and anticancer activities.

## Background

Plants have been one of the major sources of medicines since the beginning of human civilization. According to WHO (World Health Organization) data, about 80 % of the world population depends on traditional medicines based on medicinal plants [1]. These plants demonstrate effects comparable to that obtained from allopathic medicines [2]. Therefore, in recent years, research on medicinal plants has drawn enormous global attention. These plants are rich in a wide variety of phytochemicals such as tannins, terpenoids, alkaloids, flavonoids, etc., that possess diverse biological activities. Antioxidants play an important role in neutralizing free radical species, which are produced as end or by-products of normal biochemical reactions in the human body [3]. High amounts of free radical species have the potential to cause oxidative damage to DNA, lipids, and proteins, resulting in a cascade of degradative effects that contribute to human diseases such as diabetes mellitus, cancer, coronary heart disease, Alzheimer’s disease, neurodegenerative disorders, atherosclerosis, cataracts and inflammation [3]. Therefore, research on the exploration of potent natural compounds with good antioxidant activity and low cytotoxicity from plants has become an important branch of natural product chemistry.

Phytochemicals that possess free radical scavenging acitivity can be of potential use in the treatment of inflammatory disorders. Inflammation is the response of the organism to invasion by a foreign body, such as bacteria, parasites and viruses. In this context, the inflammatory response is a critical protective reaction to irritation, injury, or infection, characterized by redness, heat, swelling, loss of function and pain [4]. The understanding of the molecular and cellular mechanisms involved in the inflammatory process has increased dramatically in recent years and this has permitted the discovery of many promising phytochemicals for the development of new drugs to treat chronic inflammatory diseases, such as rheumatoid arthritis, allergy, asthma and inflammatory bowel disease. [4]. In addition, phytochemicals that scavenge free radical species can be beneficial in the treatment of cancer. Cancer is the second most common cause of death in humans standing next to cardiovascular disorders. Of this, cervical cancer is the third most common cancer and the fourth leading cause of cancer related death among women worldwide [5, 6]. Approximately 80 % of cervical cancers occur in developing countries [7]. Chemoprevention of cancer is a way of cancer control in which the occurrence of this disease is prevented by administration of one or several chemical compounds [8]. A large number of chemical compounds have been shown to prevent cancer by different mechanisms and appear to work at different stages in the neoplastic process. In recent years, there has been increasing interest in the potential cancer chemopreventive properties of phytochemicals with fewer side effects. According to literature, about 60 % of most effective anticancer/anti-infectious drugs already on the market and under clinical investigations are of products or compounds derived from natural products [9].

The coconut palm is botanically known as *Cocos nucifera* L., and is a member of the monocotyledonous family Arecaceae (Palmae) and is the only species of the genus. It is a plantation crop that grows mainly in tropical coastal areas in the Asian continent and in parts of South America and Africa [10]. The immature inflorescence of *Cocos nucifera* L. (var. aurantiaca) is used by Ayurvedic and traditional medical practitioners for the treatment of menorrhagia in Sri Lanka. Our previous studies have shown that the inflorescence of *Cocos nucifera* L. predominantly contains proanthocyanidins [11].

**Fig. 1:**
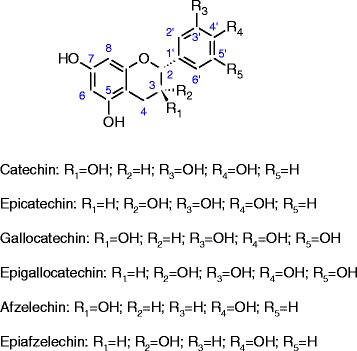
Chemical structure of flavan-3-ol units found in proanthocyanidins

Proanthocyanidins are oligomers or polymers made up of flavan-3-ol units. The most common flavan-3-ol units are (+)-catechin, (−)-epicatechin, (+)-gallocatechin and (−)-epigallocatechin while (+)-afzelechin and (−)-epiafzelechin have been reported to a lesser extent (Fig. 1) [12]. The flavan-3-ol units in proanthocyanidins are mainly linked through C4 to C8 or sometimes C4 to C6 bonds. Proanthocyanidins, which contain only these linkages, are named as B-type proanthocyanidins. When additional ether linkages are found (usually between C2 and C7), the compounds are named as A-type proanthocyanidins. A large variety of different proanthocyanidins have been reported that differ depending on the monomeric unit, substitution pattern of the monomeric unit and the extent of oligomerization [12]. Proanthocyanidins have recently attracted a considerable amount of attention in the fields of medicine, health and nutrition. They have been reported to exhibit antioxidant [13], anti-inflammatory [13], bacterial anti-adhesion [14], anticancer [15], and cardioprotective [16] activities. Today, nutritional supplements containing proanthocyanidin extracts from various plant sources are available, alone or in combination with other nutrients, as herbal extracts, capsules, or tablets [17, 18].

We have previously reported the extraction, purification and characterization of ethyl acetate soluble proanthocyanidins (EASPA) of the inflorescence of *Cocos nucifera* L. [11]. In addition, progestogenic activity of EASPA of *Cocos nucifera* L. inflorescence has also been reported in relation to its ethnomedical usage [19]. In allopathic medicine, synthetic progesterones are used to treat menorrhagia. It is significant that proanthocyanidins, are chemically different to progestogens used in allopathic medicine. Our finding suggested a possible mode of action, to explain the use of coconut inflorescence in controlling menorrhagia in Ayurveda and traditional medicine in Sri Lanka. Since inflammation is one of the mechanisms involved in menorrhagia and proanthocyanidins have been reported to mediate antioxidant and anti-inflammatory activities, it was interesting to find out whether the extracted EASPA fraction from the inflorescence of *Cocos nucifera* L. also possess antioxidant and anti-inflammatory activities. In addition, cytotoxic activity of EASPA fraction towards cervical cancer cells (HeLa cell line) which is a life threatening malignancy that originates in female reproductive tract was evaluated. Here, we report the antioxidant, anti-inflammatory and anticancer activities of the EASPA fraction of *Cocos nucifera* L. inflorescence.

## Methods

### Materials

DPPH reagent (1,1-diphenyl-2-picrylhydrazyl) was purchased from Wako Chemicals (USA, Inc.). HBSS (Hanks’ Balanced Salt Solution containing calcium chloride and magnesium chloride), gallic acid (≥98 %) and propyl gallate (≥98 %) were obtained from Sigma-Aldrich Co. (St. Louis, MO, USA). SOZ (Serum Opsonized Zymosan) was purchased from Fluka Chemie (Buchs, Switzerland). Luminol (3-aminophthalhydrazide) was obtained from Research Organics Inc. (Cleveland, OH, USA). Other chemicals and reagents were of analytical grade and purchased from Sigma-Aldrich Co. (St. Louis, MO, USA). Water when used was distilled using GFL distillation apparatus. Microplate reader (SpectraMax 340PC384) was used to measure the absorbance in antioxidant and cytotoxicity assays. Chemiluminescence in oxidative burst assay was determined by a Luminometer (Labsystems, Helsinki, Finland).

### Plant material

Inflorescences were collected from healthy adult *Cocos nucifera* L. (var. aurantiaca) palms situated in the University of Sri Jayewardenepura premises, Sri Lanka from May 2012 to April 2014. Immature inflorescence (the inflorescence which was situated just above the freshly opened inflorescence in the palm) was plucked and the spathe was removed. The inflorescence was botanically authenticated by Mr. I. U. Kariyawasam of the Department of Botany and voucher specimen (Assess. No. A3 S13, 001) was deposited in the herbarium of the Department of Botany, Faculty of Applied Sciences, University of Sri Jayewardenepura, Sri Lanka.

### Extraction and Purification of EASPA

Extraction and purification of EASPA fraction in the immature inflorescence of *Cocos nucifera* L. has already been reported. EASPA fraction of an acetone/water (7:3) extract of *Cocos nucifera* L. inflorescence was purified by chromatography on Sephadex LH-20 to yield 0.03 % of purified EASPA as an off white powder and was used for the study [11].

### Bioassays

#### Antioxidant activity

Antioxidant activity of EASPA was determined using DPPH and superoxide anion radical (SOR) scavenging assays.

##### DPPH assay

The free radical scavenging activity of the EASPA was determined using the DPPH assay as described in literature [20]. EASPA was assayed at concentrations of 1, 2, 4, 8, 16 and 32 μg/mL. Reaction mixtures containing an ethanol solution of 95 μL DPPH (300 μM) and 5 μL EASPA in DMSO were placed in a 96-well microplate and incubated at 37 °C for 30 min in the dark. The absorbance of the mixture in the 96-well plate was then measured at 517 nm. Gallic acid was used as a positive control. Negative control contained DMSO instead of the antioxidant solution while blank contained ethanol instead of DPPH solution. The IC_50_ values were calculated using the EZ-Fit Enzyme kinetics software program (Perrella Scientific Inc., Amherst, MA, USA) and compared that of the positive control. Experiments were performed in triplicate.

##### SOR scavenging assay

The SOR scavenging activity was determined according to a published method with minor modifications [21]. EASPA was assayed at concentrations of 10, 20, 30, 40, 50, 100 μg/mL. The reaction mixture contained 10 mL of EASPA in DMSO, 90 mL of phosphate buffer (0.1 M, pH 7.4), 40 mL of β-nicotanamide adenine dinucleotide (NADH) (0.2 mM) and 40 mL of nitro blue tetrazolium (NBT) (0.08 mM). The reaction was initiated by the addition of 20 mL of phenazine methosulphate (PMS) (0.008 mM). The solutions of NADH, NBT and PMS were prepared in phosphate buffer. The rate of NBT reduction was calculated from the differential absorbance at 560 nm (after 5 min of the addition of PMS) with respect to a blank solution in which PMS was replaced by buffer solution. Negative control contained DMSO instead of the antioxidant solution. Propyl gallate was used as a positive control. The IC_50_ values were calculated using the EZ-Fit Enzyme kinetics software program (Perrella Scientific Inc., Amherst, MA, USA) and compared that of the positive control. Experiments were carried out in triplicate.

#### Anti-inflammatory activity

Anti-inflammatory activity of EASPA was determined by the oxidative burst assay using chemiluminescence technique according to a published method [22]. A 25 μL cell suspension of whole blood (diluted 1:200) in HBSS was incubated with 25 μL of EASPA in HBSS at three different concentrations (1, 10 and 100 μg/mL) in a 96-well plate at 37 °C for 15 min. Medium HBSS with cell suspension was employed as a negative control. Test was carried out in white half area of 96-well plate. Incubation was performed in the thermostat chamber of Luminometer. The reactive oxygen species (ROS) production was initiated by the addition of 25 μL of SOZ in HBSS (20 mg/mL) into each well except for the blank solution. Thereafter, 25 μL of luminol in HBSS (7 × 10^5^ M) were added into each reaction mixture as well. Chemiluminescence peaks were recorded using a Luminometer in terms of relative light units (RLU). Ibuprofen was used as a positive control. The percentage of inhibition was calculated in comparison to the negative control in the maximum luminescence (the height of the peak). The results (% inhibition) were processed using the EZ-Fit Enzyme kinetics software program (Perrella Scientific Inc., Amherst, MA, USA) and compared that of the positive control. Experiments were carried out in triplicate.

#### Anticancer activity

##### Cell culture

HeLa cells (cervical cancer) and PC3 cells (prostate cancer) were cultured in Dulbecco’s Modified Eagle Medium (DMEM) containing 5 % fetal bovine serum (FBS), 100 IU/mL penicillin and 100 μg/mL streptomycin. The cells were incubated at 37 °C in humidified atmosphere of 5 % CO_2_ in the air.

### Cytotoxicity assay

MTT (3-[4,5-dimethylthiazole-2-yl]-2,5-diphenyl tetrazolium bromide) colorimetric assay was used to determine the cytotoxicity of EASPA to both HeLa cells and PC3 according to a previously reported method [23]. The HeLa cells were seeded onto a 96-well plate at a concentration of 1 × 10^5^ cells/mL (100 μL/well) in DMEM culture medium and cultured at 37 °C for 24 h. Subsequently, the culture medium was replaced with 200 μL of fresh DMEM containing EASPA at different concentrations (5, 20, 80 μg/mL) and incubated for 48 h. The DMEM was employed as a negative control. Thereafter, 200 μL MTT solution (0.5 mg/mL) in phosphate buffered saline was added to each well, and the plate was incubated for 4 h. The solutions in each well were then removed, and the remaining formazan crystals in the cells were dissolved with 100 μL of DMSO. The absorbance of the mixture in the 96-well plate was then measured using a microplate reader at 570 nm. The cytotoxicity of EASPA at different concentrations was calculated as the percentage of the absorbance relative to that of the negative control. Tamoxifen was used as a positive control. Experiments were performed in triplicate. The results (% inhibition) were processed using Soft- Max Pro software (Molecular Device, USA).

The same procedure was adapted for PC3 cells as well. Doxorubicin was used as a positive control.

### Statistical analysis

The results are represented as the IC_50_ ± SEM. Every statistical analysis was performed with one-way ANOVA, followed by student *T* test using Minitab 17.0 software. Differences were accepted as statistically significant at *P* ≤ 0.05.

## Results and discussion

### Antioxidant activity of EASPA

The antioxidant activity of EASPA was evaluated by their ability to scavenge DPPH and superoxide radicals. The DPPH radical scavenging activity has been extensively used to determine the antioxidant capacity of phenolic compounds. DPPH is a nitrogen centered stable free radical, and their color changes from violet to yellow when it is reduced by the process of either hydrogen or electron donation [24]. Superoxide anion radicals are produced by a number of cellular reactions, including various enzyme systems, such as lipoxygenases, peroxidase, NADPH oxidase and xanthine oxidase [21]. Superoxide anion is a weak oxidant. However, it plays important roles in the formation of powerful and dangerous ROS such as hydrogen peroxide, hydroxyl radical, and singlet oxygen, which induce oxidative damage in lipids, proteins, and DNA. In the present study, superoxide radicals were generated in a non-enzymatic system (PMS-NADH) and quantified by the spectrophotometric measurement of the reduction product of NBT [21]. Results of antioxidant assays are given in Table 1. EASPA (IC_50_ = 11.02 ± 0.60 μg/mL) showed lesser radical scavenging activity than standard gallic acid (IC_50_ = 4.30 ± 0.43 μg/mL) against DPPH radicals. Further, EASPA (IC_50_ = 26.11 ± 0.72 μg/mL) showed lower antioxidant activity compared to standard propyl gallate (IC_50_ = 22.56 ± 0.56 μg/mL) against superoxide radicals. Gallic acid and propyl gallate are stronger antioxidants than ascorbic acid, which is the most commonly used standard for antioxidant assays [25]. According to these results, EASPA possesses antioxidant activity but not as much as the standards used.

**Table 1:** Antioxidant activity of the EASPA from the inflorescence of *C. nucifera* L

Antioxidant assay	EASPA (μg/mL)	Standard (μg/mL)
DPPH assay	11.02 ± 0.60	4.30 ± 0.43 (Gallic acid)
SOR scavenging assay	26.11 ± 0.72	22.56 ± 0.56 (Propyl gallate)

All values are presented as IC_50_ ± SEM, *n* = 3

### Anti-inflammatory activity of EASPA

Anti-inflammatory activity of EASPA was determined by oxidative burst assay using chemiluminescence technique. The oxidative burst is an important step in bacterial killing and involves a series of metabolic events that take place when phagocytes are stimulated, resulting in the production of superoxide, hydrogen peroxide, and other more potent oxidizing radicals. Chemiluminescence is based on the amplification of natural luminescence emitted when ROS are released during phagocytosis. All the ROS convert chemiluminogenic probes, as luminol, into the aminophtalate anion, which is inherently unstable and its return to the ground state is accompanied by the release of photons. This light production is one of the characteristics of the oxidative burst reaction [26]. The results of the oxidative burst assay are shown in Table 2. Results indicate that the EASPA (IC_50_ = 10.31 ± 1.11 μg/mL) exhibits similar (P ≥ 0.05) anti-inflammatory activity than ibuprofen (IC_50_ = 11.20 ± 1.90 μg/mL), which is one of the most widely used drugs for inflammatory diseases.

**Table 2:** Anti-inflammatory activity of EASPA from inflorescence of *Cocos nucifera* L

Anti-inflammatory assay	EASPA (μg/mL)	Ibuprofen (μg/mL)
Oxidative burst assay	10.31 ± 1.11	11.20 ± 1.90

All values are presented as IC_50_ ± SEM, *n* = 3

### Cytotoxicity of EASPA towards HeLa and PC3 cell lines

Cytotoxicity was measured by the MTT assay based on the mitochondrial reduction of a tetrazolium bromide salt, which turns into purple formazan crystals by viable and metabolic active cells [23]. The MTT assay is a widely used method for the evaluation of cytotoxic activity. The results of the MTT assay are given in the Table 3. According to the results, EASPA (IC_50_ = 18.78 ± 0.90 μg/mL) exhibited higher cytotoxicity (*P* ≤ 0.05) compared to tamoxifen (IC_50_ = 28.80 ± 1.94 μg/mL) against the HeLa cell line, while low cytotoxicity (IC_50_ = 44.21 ± 0.73 μg/mL) was shown towards the PC3 cell line compared to doxorubicin (IC_50_ = 1.38 ± 0.16 μg/mL). Tamoxifen is commonly used positive control for HeLa cell line [27]. Thus, according to the results EASPA mediate cytotoxic activity against HeLa cells.

**Table 3:** Cytotoxic activity of EASPA from inflorescence of *Cocos nucifera* L. towards HeLa and PC3 cell lines

Type of cell line	EASPA (μg/mL)	Standard (μg/mL)
Hela cell line PC3 cell line	18.78 ± 0.90* 44.20 ± 0.73	28.80 ± 1.94 (Tamoxifen)* 1.38 ± 0.16 (Doxorubicin)

All values are presented as IC_50_ ± SEM, *n* = 3, *P ≤ 0.05

## Conclusion

The EASPA fraction showed antioxidant and anti-inflammatory activities. These activities of EASPA may also play an important role in treating menorrhagia with the use of the inflorescence of *Cocos nucifera* L. in Ayurveda. In addition, EASPA showed potent cytotoxic activity towards HeLa cell line. Our findings open the possibility of clinical use of a new class of drug, which can be used to treat menorrhagia and cervical cancer in women.

## Abbreviations

DMEM: Dulbecco’s modified eagle medium
DPPH: 1,1-diphenyl-2-picrylhydrazyl
EASPA: Ethyl acetate soluble proanthocyanidins
FBS: Fetal bovine serum
HBSS: Hanks’ Balanced Salt Solution containing calcium chloride and magnesium chloride
MTT: 3-[4,5-dimethylthiazole-2-yl]-2,5-diphenyl tetrazolium bromide
NADH: β-nicotanamide adenine dinucleotide
NBT: Nitro blue tetrazolium
PMS: Phenazine methosulphate
RLU: Relative light units
ROS: Reactive oxygen species
SOR: Superoxide anion radical
SOZ: Serum opsonized zymosan
WHO: World health organization
